# Pathologically relevant aldoses and environmental aldehydes cause cilium disassembly via formyl group-mediated mechanisms

**DOI:** 10.1093/jmcb/mjad079

**Published:** 2023-12-06

**Authors:** Te Li, Min Liu, Fan Yu, Song Yang, Weiwen Bu, Kai Liu, Jia Yang, Hua Ni, Mulin Yang, Hanxiao Yin, Renjie Hong, Dengwen Li, Huijie Zhao, Jun Zhou

**Affiliations:** Haihe Laboratory of Cell Ecosystem, State Key Laboratory of Medicinal Chemical Biology, Tianjin Key Laboratory of Protein Science, College of Life Sciences, Nankai University, Tianjin 300071, China; Laboratory of Tissue Homeostasis, Haihe Laboratory of Cell Ecosystem, Tianjin 300462, China; Haihe Laboratory of Cell Ecosystem, State Key Laboratory of Medicinal Chemical Biology, Tianjin Key Laboratory of Protein Science, College of Life Sciences, Nankai University, Tianjin 300071, China; Haihe Laboratory of Cell Ecosystem, State Key Laboratory of Medicinal Chemical Biology, Tianjin Key Laboratory of Protein Science, College of Life Sciences, Nankai University, Tianjin 300071, China; Haihe Laboratory of Cell Ecosystem, State Key Laboratory of Medicinal Chemical Biology, Tianjin Key Laboratory of Protein Science, College of Life Sciences, Nankai University, Tianjin 300071, China; Haihe Laboratory of Cell Ecosystem, State Key Laboratory of Medicinal Chemical Biology, Tianjin Key Laboratory of Protein Science, College of Life Sciences, Nankai University, Tianjin 300071, China; Haihe Laboratory of Cell Ecosystem, State Key Laboratory of Medicinal Chemical Biology, Tianjin Key Laboratory of Protein Science, College of Life Sciences, Nankai University, Tianjin 300071, China; Haihe Laboratory of Cell Ecosystem, State Key Laboratory of Medicinal Chemical Biology, Tianjin Key Laboratory of Protein Science, College of Life Sciences, Nankai University, Tianjin 300071, China; Haihe Laboratory of Cell Ecosystem, State Key Laboratory of Medicinal Chemical Biology, Tianjin Key Laboratory of Protein Science, College of Life Sciences, Nankai University, Tianjin 300071, China; Haihe Laboratory of Cell Ecosystem, State Key Laboratory of Medicinal Chemical Biology, Tianjin Key Laboratory of Protein Science, College of Life Sciences, Nankai University, Tianjin 300071, China; Haihe Laboratory of Cell Ecosystem, State Key Laboratory of Medicinal Chemical Biology, Tianjin Key Laboratory of Protein Science, College of Life Sciences, Nankai University, Tianjin 300071, China; Haihe Laboratory of Cell Ecosystem, State Key Laboratory of Medicinal Chemical Biology, Tianjin Key Laboratory of Protein Science, College of Life Sciences, Nankai University, Tianjin 300071, China; Center for Cell Structure and Function, Collaborative Innovation Center of Cell Biology in Universities of Shandong, Shandong Provincial Key Laboratory of Animal Resistance Biology, College of Life Sciences, Shandong Normal University, Jinan 250014, China; Haihe Laboratory of Cell Ecosystem, State Key Laboratory of Medicinal Chemical Biology, Tianjin Key Laboratory of Protein Science, College of Life Sciences, Nankai University, Tianjin 300071, China; Center for Cell Structure and Function, Collaborative Innovation Center of Cell Biology in Universities of Shandong, Shandong Provincial Key Laboratory of Animal Resistance Biology, College of Life Sciences, Shandong Normal University, Jinan 250014, China

**Keywords:** carbohydrate metabolism disorder, aldose, aldehyde, cilium disassembly, formyl group, calcium influx, HDAC6

## Abstract

Carbohydrate metabolism disorders (CMDs), such as diabetes, galactosemia, and mannosidosis, cause ciliopathy-like multiorgan defects. However, the mechanistic link of cilia to CMD complications is still poorly understood. Herein, we describe significant cilium disassembly upon treatment of cells with pathologically relevant aldoses rather than the corresponding sugar alcohols. Moreover, environmental aldehydes are able to trigger cilium disassembly by the steric hindrance effect of their formyl groups. Mechanistic studies reveal that aldehydes stimulate extracellular calcium influx across the plasma membrane, which subsequently activates the calmodulin–Aurora A–histone deacetylase 6 pathway to deacetylate axonemal microtubules and triggers cilium disassembly. *In vivo* experiments further show that *Hdac6* knockout mice are resistant to aldehyde-induced disassembly of tracheal cilia and sperm flagella. These findings reveal a previously unrecognized role for formyl group-mediated cilium disassembly in the complications of CMDs.

## Introduction

Cilia are microtubule-based, antenna-like organelles that protrude from the cell surface. Primary cilia are able to sense extracellular signals primarily through receptor proteins on the ciliary membrane (Nag and Resnick, 2017; Lee, 2020; Qi and Zhou, 2021; Yang et al., 2021), whereas motile cilia are critically involved in fluid flow and cell motility (Hirokawa et al., 2006). Dysfunction of cilia due to genetic mutations leads to a group of diseases, collectively called ciliopathies. Ciliopathies usually involve multiorgan defects, such as polydactyly, infertility, obesity, retinal degeneration, and polycystic kidney disease. In addition, defects in primary cilia have been implicated in the development of cancer and a number of other diseases (Liu et al., 2018; Higgins et al., 2019; Tian et al., 2023; Wu et al., 2023).

Microtubules assembled from α/β-tubulin heterodimers are the major structural components of ciliary axonemes (Nachury and Mick, 2019). These microtubules accumulate several highly conserved post-translational modifications, such as acetylation, glutamylation, and detyrosination (L’Hernault and Rosenbaum, 1983; LeDizet and Piperno, 1987; Wloga et al., 2017). The acetylation of α-tubulin has been demonstrated to have microtubule lattice-stabilizing activity and modulate the sensitivity to microtubule drugs (Akella et al., 2010; Cueva et al., 2012; Kim et al., 2013). Histone deacetylase 6 (HDAC6), the major deacetylase for α-tubulin, is able to destabilize the microtubule lattice, and overexpression of HDAC6 promotes cilium disassembly, leading to fewer and shortened cilia (Yang et al., 2014; Bangs et al., 2015). Phosphorylation of HDAC6 by apoptosis signal-regulating kinase 1 has been reported to accumulate in retinal photoreceptors and cause the disassembly of photoreceptor cilia (Ran et al., 2020). In contrast, *Hdac6*-deficient mice have normal cilia without any significant ciliopathy-related symptoms (Zhang et al., 2008; Plotnikova et al., 2012).

Ciliary defects have recently been implicated in the complications of diabetes, a group of carbohydrate metabolism disorders (CMDs) characterized by high blood glucose levels (Volta and Gerdes, 2017; Kluth et al., 2019; Panchapakesan and Pollock, 2020; Chinipardaz et al., 2021). Specifically, *O*-linked β-*N*-acetylglucosaminylation (*O*-GlcNAcylation), a post-translational modification associated with diabetes, has been identified as a critical factor causing ciliary defects, and targeting the *O*-GlcNAcylation–cilium axis could partially alleviate the retinal dysfunction associated with diabetes (Yu et al., 2019, 2020). In addition, galactosemia, a CMD characterized by the inability to convert galactose to glucose, also leads to severe complications such as retinopathy and nephropathy (McCorvie and Timson, 2011; Ozgun et al., 2019; Welsink-Karssies et al., 2020). Alpha-mannosidosis, another CMD characterized by the lysosomal accumulation of small mannose-rich oligosaccharides, results in skeletal abnormalities, hearing impairment, and several other symptoms. The high similarity in clinical symptoms between CMD complications and ciliopathies suggests that ciliary defects may contribute to the complications of CMDs.

Three monosaccharides (glucose, galactose, and mannose) associated with the aforementioned CMDs have a common feature in that all of them are aldoses (i.e. containing formyl groups), but it is unclear whether the formyl groups are involved in the ciliopathy-like complications of these CMDs. Interestingly, environmental aldehydes are known to affect the structure and function of cilia, especially the motile cilia of the respiratory tract (Bansal et al., 2011; Xiong et al., 2018). Short-term exposure to aldehydes, such as formaldehyde and acrolein, can cause lung discomfort, severe lung edema, and even death, and long-term exposure to aldehydes may lead to the development of cancer. In addition, chloral hydrate (CH), an aldehyde derivative, is able to induce a breakdown of the motile cilia of protozoa (Sanders and Salisbury, 1994) and has been used to remove primary cilia from mammalian cells (Delaine-Smith et al., 2014; Coughlin et al., 2016). However, the mechanisms by which deciliation occurs in response to aldehydes and their derivatives remain elusive. In this study, we provide the first evidence that both pathologically relevant aldoses and environmental aldehydes induce cilium disassembly via formyl group-mediated mechanisms.

## Results

### Pathologically relevant aldoses cause cilium disassembly in a formyl group-dependent manner

**Figure 1:**
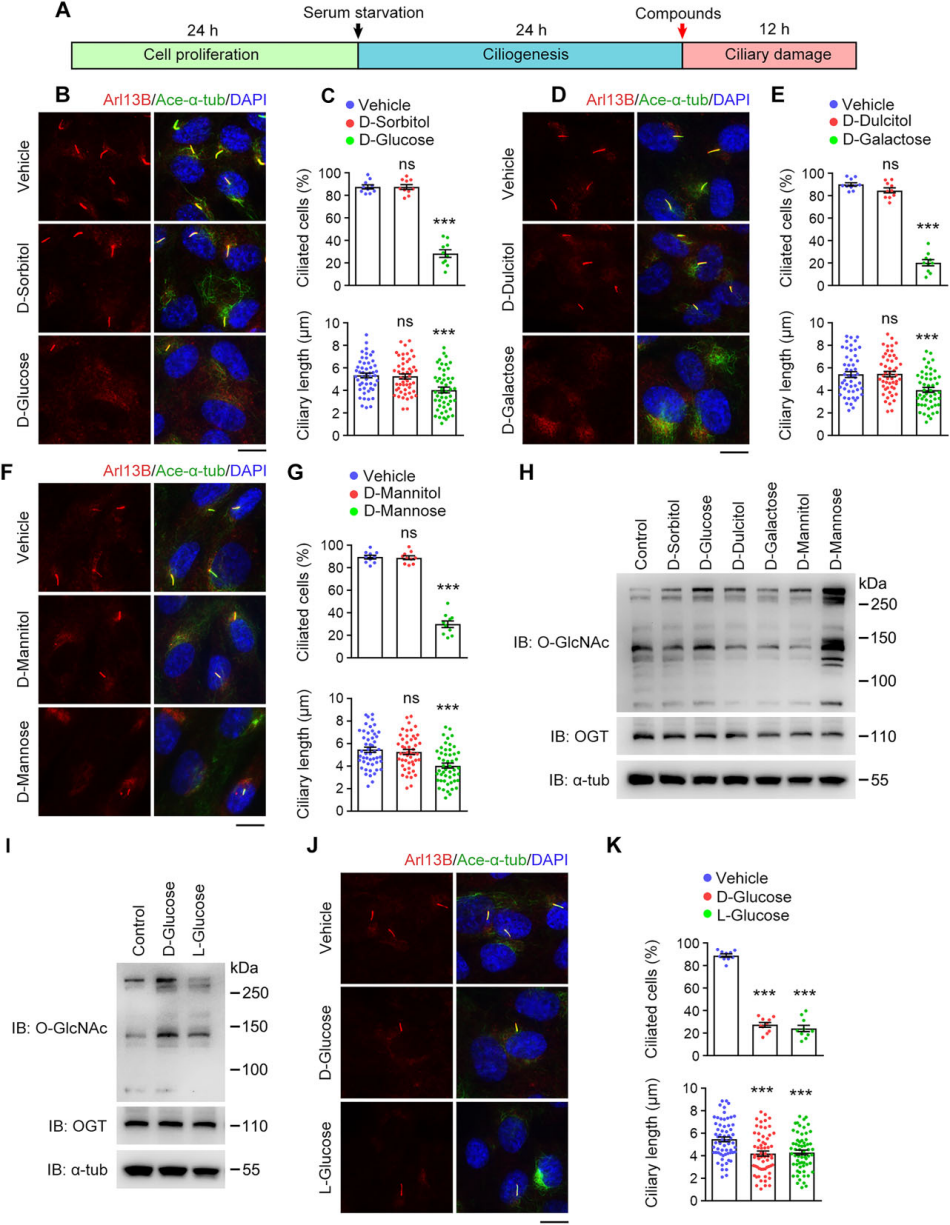
Aldoses trigger cilium disassembly in a formyl group-dependent manner. (**A**) Strategy used to study the effects of aldoses on cilia. Ciliated RPE-1 cells were incubated with aldoses for 12 h, followed by quantitative analysis for the percentage of ciliated cells and ciliary length. (**B**–**K**) RPE-1 cells were incubated with vehicle (PBS) or the indicated compounds (25 mM) in serum-free medium. (**B, D, F**, and **J**) Immunofluorescence images of ciliated cells stained for Arl13B, acetylated α-tubulin, and 4′,6-diamidino-2-phenylindole(DAPI). Scale bar, 10 μm. (**C, E, G**, and **K**) The percentage of ciliated cells (*n* = 10 fields from three independent experiments) and quantification of ciliary length (*n* = 50 cilia from three independent experiments). (**H** and **I**) Immunoblotting for *O*-GlcNAc, *O*-GlcNAc transferase (OGT), and αtubulin. Data are presented as mean ± SEM. An unpaired two-tailed *t*-test was performed. ***P < 0.001; ns, not significant.

To test our hypothesis that ciliary defects may contribute to the complications of CMDs, we examined the effects of D-glucose, D-galactose, and D-mannose on primary cilia of human retinal pigment epithelial-1 (RPE-1) cells. Following serum starvation, each of these aldoses was added to the culture medium at the pathologically relevant concentration (Figure 1A; Stoner, 2017; Mathew et al., 2023). To exclude the effect of osmolarity changes, sorbitol, dulcitol, or mannitol at the same concentration, respectively, was used as the control. Notably, aldose treatment significantly decreased the proportion of ciliated cells and ciliary length, while none of the control sugar alcohols caused ciliary defects (Figure 1B–G). Consistently, xylose, a monosaccharide of the aldopentose type, significantly disrupted cilia, while xylitol did not have any obvious effect (Supplementary Figure S1A–C). Considering the structural difference between these aldoses and sugar alcohols (Supplementary Figure S1D), we hypothesized that the formyl groups of aldoses may be responsible for the disassembly of cilia. To test this hypothesis, fructose, which has a molecular formula identical to that of glucose but lacks the formyl group, was examined. As expected, fructose treatment had no effect on cilia (Supplementary Figure S1E and F). Collectively, these results indicate that the formyl groups of aldoses cause cilium disassembly under pathologically relevant conditions.

Given the known function of *O*-GlcNAcylation in regulating ciliogenesis and ciliary length (Yu et al., 2019; Yang et al., 2022), we investigated whether aldose treatment affects the global *O*-GlcNAcylation level in RPE-1 cells. Immunoblotting showed that D-mannose dramatically increased the *O*-GlcNAcylation level, whereas the control sugar alcohol slightly decreased *O*-GlcNAcylation (Figure 1H). In contrast, both D-glucose and D-galactose showed similar effects on the global *O*-GlcNAcylation level as their control sugar alcohols (Figure 1H). These results suggest that *O*-GlcNAcylation may not be a critical factor in the formyl group-dependent disassembly of cilia.

Since L-glucose is rarely taken up into mammalian cells and cannot be metabolized (Pitt et al., 2021), this sugar was introduced into our assay to further evaluate the roles of the formyl group and *O*-GlcNAcylation in the disassembly of cilia. Immunoblotting demonstrated that L-glucose had weaker activity in elevating the global *O*-GlcNAcylation level in RPE-1 cells than D-glucose (Figure 1I). This result is consistent with the fact that L-glucose cannot be converted to uridine diphosphate-GlcNAc to promote *O*-GlcNAcylation. However, L-glucose treatment decreased the proportion of ciliated cells and ciliary length at a comparable level to D-glucose treatment (Figure 1J and K), confirming that the formyl group itself, instead of the subsequent *O*-GlcNAcylation, mediates the cilium disassembly activity of aldoses.

### Steric hindrance of the formyl groups contributes to the deciliating activity of aldehydes

**Figure 2:**
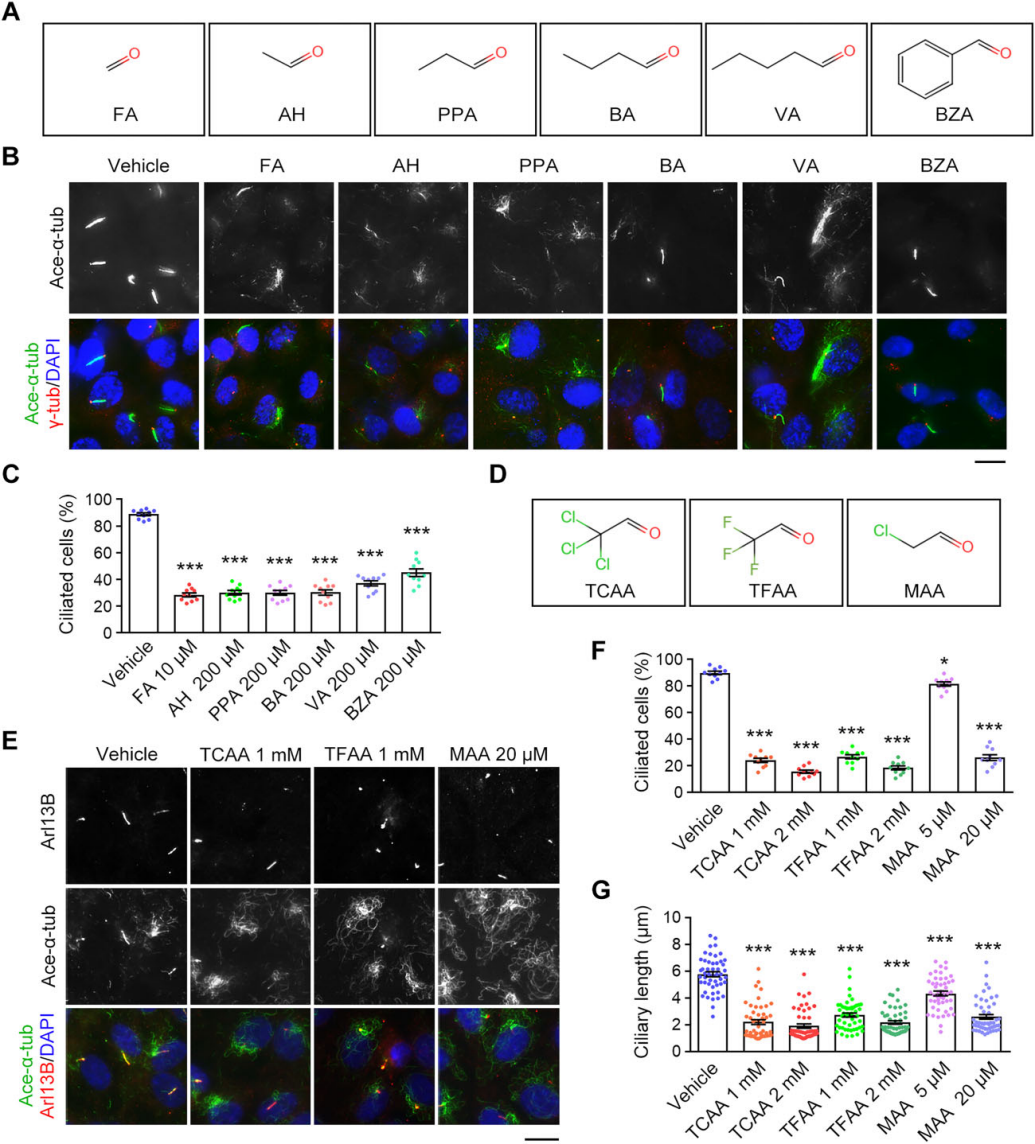
The cilium disassembly activity of aldehydes is regulated by steric hindrance of the formyl groups. (**A**) Chemical structures of the aldehydes used in this study. FA, formaldehyde; AH, acetaldehyde; PPA, propionaldehyde; BA, butyraldehyde; VA, valeraldehyde; BZA, benzaldehyde. (**B** and **C**) RPE-1 cells were incubated with vehicle (DMSO) or the indicated aldehydes in serum-free medium. (**B**) Immunofluorescence images of ciliated cells stained for γ-tubulin, acetylated α-tubulin, and DAPI. Scale bar, 10 μm. (**C**) Quantification of the percentage of ciliated cells (*n* = 10 fields from 3 independent experiments). (**D**) Chemical structures of trichloroacetaldehyde (TCAA) and its analogues trifluoroacetaldehyde (TFAA) and monochloroacetaldehyde (MAA). (**E**–**G**) RPE-1 cells were incubated with vehicle (DMSO) or the indicated aldehydes in serum-free medium. (**E**) Immunofluorescence images of ciliated cells stained for Arl13B, acetylated α-tubulin, and DAPI. Scale bar, 10 μm. (**F** and **G**) The percentage of ciliated cells (*n* = 10 fields from three independent experiments) and quantification of ciliary length (*n* = 50 cilia from three independent experiments). Data are presented as mean ± SEM. An unpaired two-tailed *t*-test was performed. *P < 0.05; ***P < 0.001; ns, not significant.

Our finding that aldoses (polyhydroxy aldehydes) induce cilium disassembly prompted us to examine whether other aldehydes possess the similar activity. We analyzed five aliphatic aldehydes, including formaldehyde, acetaldehyde, propionaldehyde, butyraldehyde, and valeraldehyde, and an aromatic aldehyde, benzaldehyde. All these aldehydes significantly decreased the proportion of ciliated cells and ciliary length (Figure 2A–C). Interestingly, the ability of aldehydes to cause cilium disassembly gradually decreased with the increasing number of carbon atoms in the aldehydes (Figure 2C). Aldehydes have been shown to form adducts with phospholipid molecules, proteins, and DNAs in a covalent manner (LoPachin and Gavin, 2014). Our results thus indicate that steric effects may modulate aldehyde binding to phospholipids or proteins on the cell surface.

To further investigate the potential steric effect of aldehydes on cilium disassembly, we used three derivatives of acetaldehyde, including trichloroacetaldehyde, trifluoroacetaldehyde, and monochloroacetaldehyde, in which the methyl hydrogens were replaced with chloro or fluoro groups (Figure 2D). Strikingly, monochloroacetaldehyde, which has the least steric hindrance of the formyl group, induced cilium disassembly at a much lower concentration than the other two aldehydes (Figure 2E–G). Next, we treated ciliated RPE-1 cells with trichloroacetic acid and trichloroethanol to examine the effect of the carboxyl group (Supplementary Figure S2A). Neither trichloroacetic acid nor trichloroethanol induced cilium disassembly, whereas trichloroacetaldehyde showed a remarkable effect (Supplementary Figure S2B–D). Collectively, these results suggest that steric hindrance of the formyl groups may mediate the activity of aldehydes to induce cilium disassembly.

### Aldehyde-induced cilium disassembly is reversible and results from the deacetylation of axonemal microtubules

**Figure 3:**
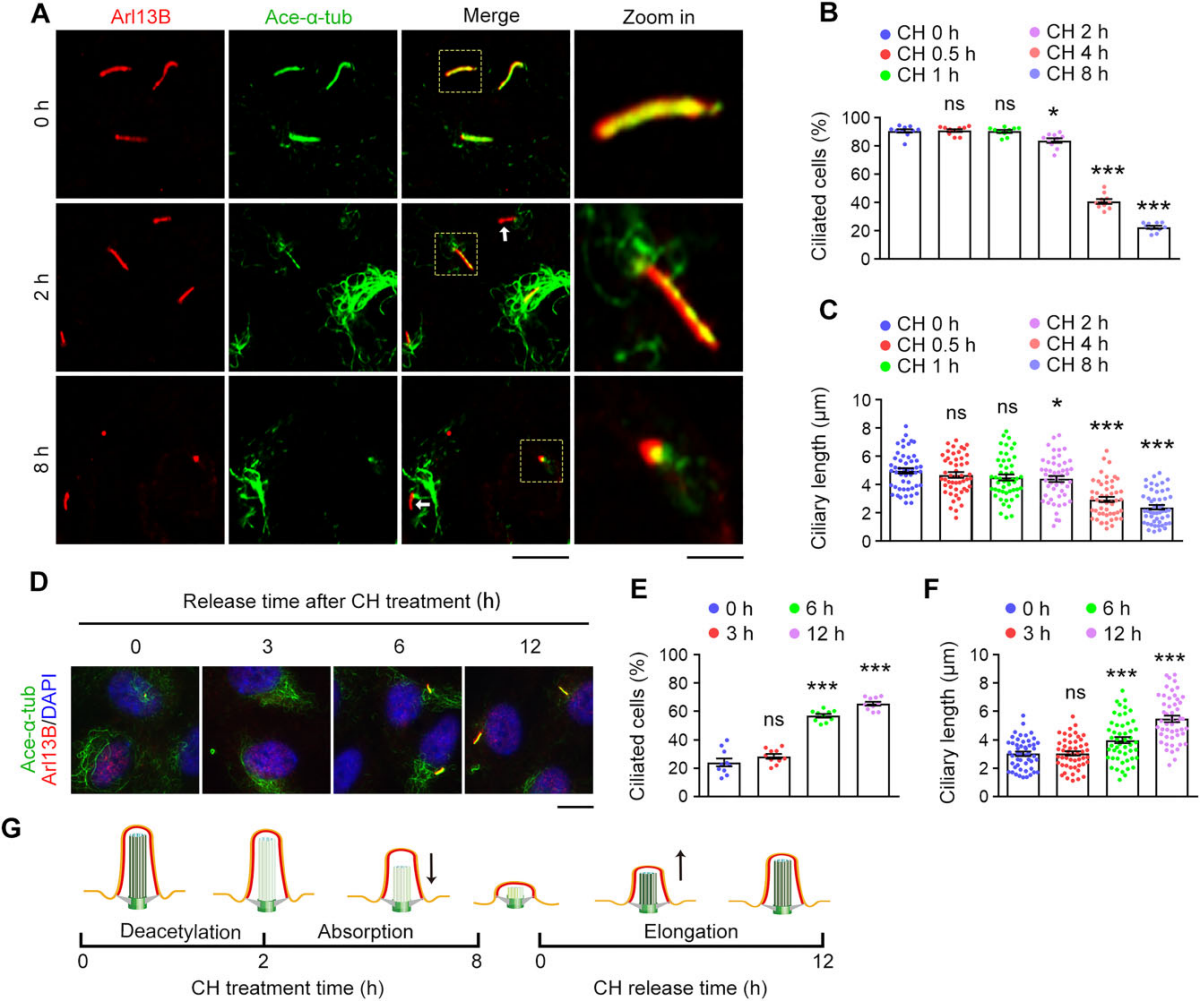
Aldehydes cause cilium disassembly by decreasing the acetylation and stability of axonemal microtubules. (**A**–**C**) RPE-1 cells were incubated with CH in serum-free medium for the indicated time. (**A**) Immunofluorescence images of ciliated cells stained for Arl13B and acetylated α-tubulin. Arrows indicate Arl13B-positive but acetylated α-tubulin-negative cells. Scale bar, 10 μm (original) or 2.5 μm (zoomed). (**B** and **C**) The percentage of ciliated cells (*n* = 10 fields from three independent experiments) and quantification of ciliary length (*n* = 50 cilia from three independent experiments). (**D**–**F**) RPE-1 cells were released from CH treatment and cultured in serum-free medium for the indicated time. (**D**) Immunofluorescence images of ciliated cells stained for Arl13B, acetylated α-tubulin, and DAPI. Scale bar, 10 μm. (**E** and **F**) The percentage of ciliated cells (*n* = 10 fields from three independent experiments) and quantification of ciliary length (*n* = 50 cilia from three independent experiments). (**G**) Schematic illustration of ciliary changes in response to CH treatment and CH removal. Data are presented as mean ± SEM. An unpaired two-tailed *t*-test was performed. *P < 0.05; ***P < 0.001; ns, not significant.

Trichloroacetaldehyde readily reacts with water to form CH (Supplementary Figure S3A), which has been used extensively to remove both primary and motile cilia (Kennedy and Brittingham, 1968; Chakrabarti et al., 1998; Praetorius and Spring, 2003). For motile cilia, CH induces ciliary loss by breaking down the axoneme from the basal body (Chakrabarti et al., 1998). However, little is known about how CH induces the removal of primary cilia. We treated ciliated RPE-1 cells with CH and then analyzed the cilium disassembly process at multiple time points. Interestingly, we observed a dramatic decrease in the acetylation of ciliary axonemal microtubules after 2 h of CH treatment (Figure 3A). After 8 h of CH treatment, most primary cilia displayed short dysmorphic stump-like structures, with a few remaining short primary cilia, as indicated by ciliary membrane-associated protein Arl13B, completely losing acetylated α-tubulin (Figure 3A). Quantitative analysis showed that the percentage of ciliated cells slightly decreased after 2 h of CH treatment, which coincided with a dramatic reduction in the acetylation of ciliary axonemal microtubules and significantly dropped after 4–8 h of CH treatment, along with a significant reduction in ciliary length (Figure 3B and C). Given the presence of multiple post-translational modifications of axonemal microtubules (Wloga et al., 2017), we asked whether CH treatment affects the polyglutamylation and detyrosination of ciliary tubulin. Interestingly, neither tubulin polyglutamylation nor detyrosination was influenced by CH treatment (Supplementary Figure S3B–F).

We next examined whether the cilium disassembly induced by aldehydes is reversible. RPE-1 cells were treated with CH for 12 h to cause deciliation, and then the cells were released from CH treatment and subsequently subjected to immunostaining to analyze cilium regeneration at different time points. We found that after CH was withdrawn from the culture medium, both the percentage of ciliated cells and ciliary length gradually increased over time (Figure 3D–F), indicating that aldehyde-induced cilium disassembly is indeed reversible.

These results suggest that aldehyde-induced disassembly of primary cilia is accomplished by the deacetylation/destabilization and shortening of axonemal microtubules, followed by ciliary membrane absorption (Figure 3G). This deciliating mechanism is clearly different from the severing mechanism observed for the disassembly of motile cilia.

### Aldehyde-induced calcium influx acts upstream of axonemal microtubule deacetylation

**Figure 4:**
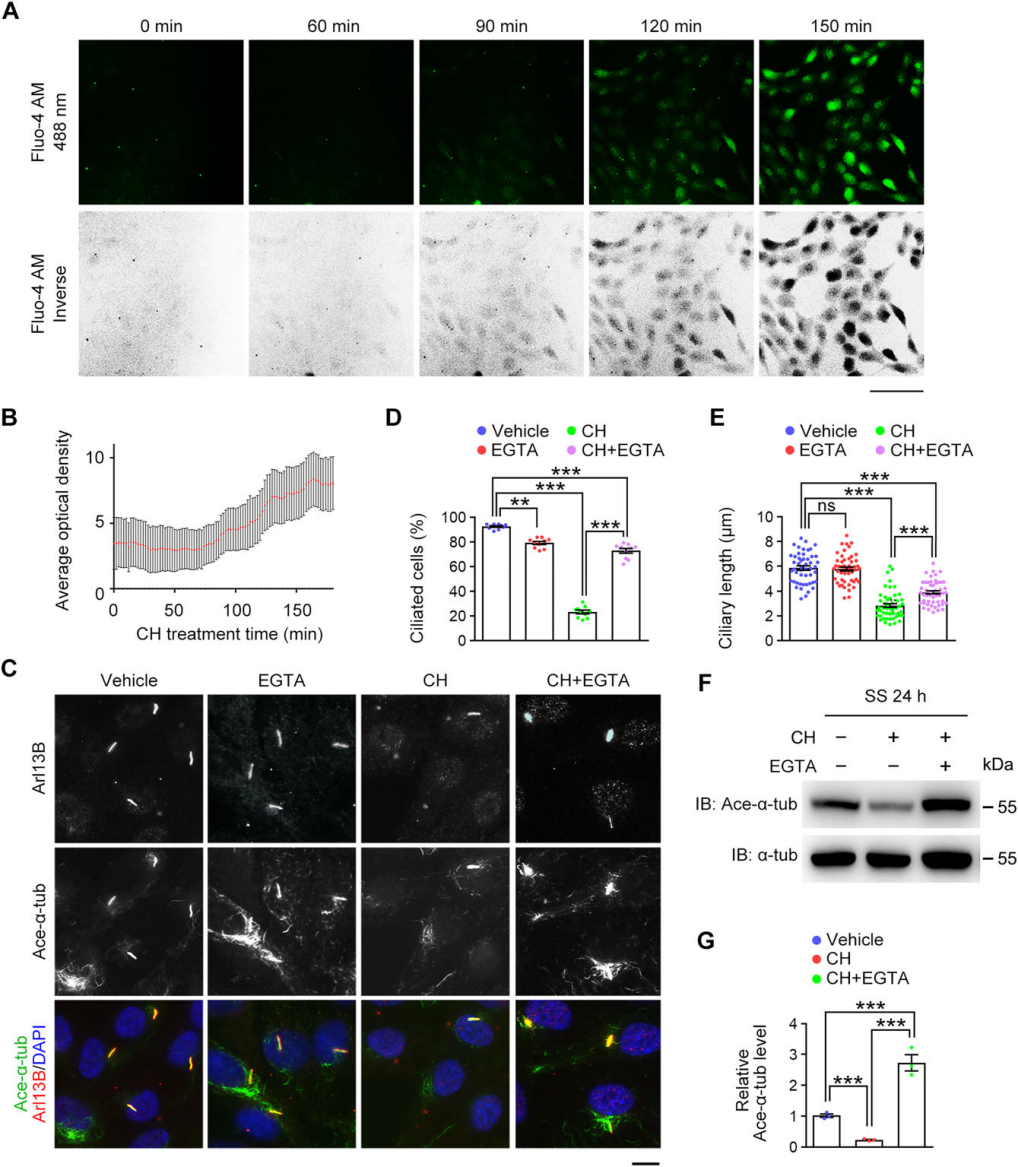
Aldehydes stimulate calcium influx to trigger axonemal microtubule deacetylation. (**A** and **B**) RPE-1 cells were incubated with CH in serum-free medium for the indicated time. (**A**) Live-cell tracking fluorescence images of calcium influx. Scale bar, 100 μm. (**B**) Quantification of calcium influx (*n* = 10 cells for each time point). The fluorescence intensity was normalized to the background at each time point. (**C**–**E**) RPE-1 cells were incubated with vehicle (PBS), EGTA (0.5 mM), CH (1 mM), or CH (1 mM) plus EGTA (0.5 mM) in serum-free medium for ∼120 min. (**C**) Immunofluorescence images of ciliated cells stained for Arl13B, acetylated α-tubulin, and DAPI. Scale bar, 10 μm. (**D** and **E**) The percentage of ciliated cells (*n* = 10 fields from three independent experiments) and quantification of ciliary length (*n* = 50 cilia from three independent experiments). (**F** and **G**) RPE-1 cells were incubated with vehicle (PBS), CH (1 mM), or CH (1 mM) plus EGTA(5 μM) in serum-free medium. (**F**) Immunoblotting for α-tubulin and acetylated α-tubulin. (**G**) Quantification of the level of acetylated α-tubulin relative to α-tubulin (*n* = 3 independent experiments). Data are presented as mean ± SEM. An unpaired two-tailed *t*-test was performed. **P < 0.01; ***P < 0.001; ns, not significant.

Aldehydes have been demonstrated to induce the influx of extracellular calcium into cells through calcium channels (McNamara et al., 2007; Fischer et al., 2014; Samak et al., 2016; Shang et al., 2016), and inhibition of calcium influx can rescue mitotic defects induced by CH treatment (Lee et al., 1987). Therefore, we investigated whether calcium influx occurs during aldehyde-induced cilium disassembly. The calcium probe Fluo-4 AM was utilized to track intracellular calcium in ciliated RPE-1 cells. We observed that the intracellular calcium concentration started to increase after CH treatment for ∼90 min (Figure 4A and B). Interestingly, CH-induced cilium disassembly started ∼120 min after CH treatment (Figure 4A–C).

To test whether calcium influx plays a role in aldehyde-induced cilium disassembly, we used ionomycin, a calcium carrier, to elevate the intracellular calcium concentration, mimicking the effect of CH treatment. Immunostaining revealed that ionomycin treatment also caused cilium disassembly (Supplementary Figure S4A and B). To further analyze the role of calcium influx in aldehyde-induced deciliation, the culture medium was pretreated with ethylene glycol-bis(beta-aminoethyl ether)-*N,N,N*′,*N*′-tetraacetic acid (EGTA) to deplete calcium and then used for the treatment of RPE-1 cells with CH. We observed that CH-induced cilium disassembly was significantly reversed by using EGTA-pretreated medium (Figure 4C–E). We then investigated whether EGTA-mediated calcium depletion affects the activity of CH to cause microtubule deacetylation. Immunostaining and immunoblotting demonstrated that calcium depletion by EGTA significantly restored the acetylation of both axonemal and total cellular microtubules in CH-treated cells (Figure 4C, F, and G).

We next examined whether the effect of aldehyde-induced calcium influx on cilium disassembly relies on the presence of cilia. To simultaneously visualize cilia and calcium influx, RPE-1 cells expressing tRFP-tagged Smoothened (Smo-tRFP) were serum-starved to induce cilium formation and live imaged (Lu et al., 2015). Consistently, re-adding serum together with CH treatment led to a dramatic increase in calcium signals (Supplementary Figure S5A and B). However, there was no obvious difference in the calcium signals between ciliated and non-ciliated cells after CH treatment (Supplementary Figure S5A and B), suggesting that the aldehyde-induced activation of calcium signals is independent of cilia. Thus, these findings indicate that calcium influx induced by aldehydes is critical for them to trigger the deacetylation and destabilization of axonemal microtubules.

### Aldehydes activate the calmodulin–Aurora A–HDAC6 pathway to cause cilium disassembly

**Figure 5:**
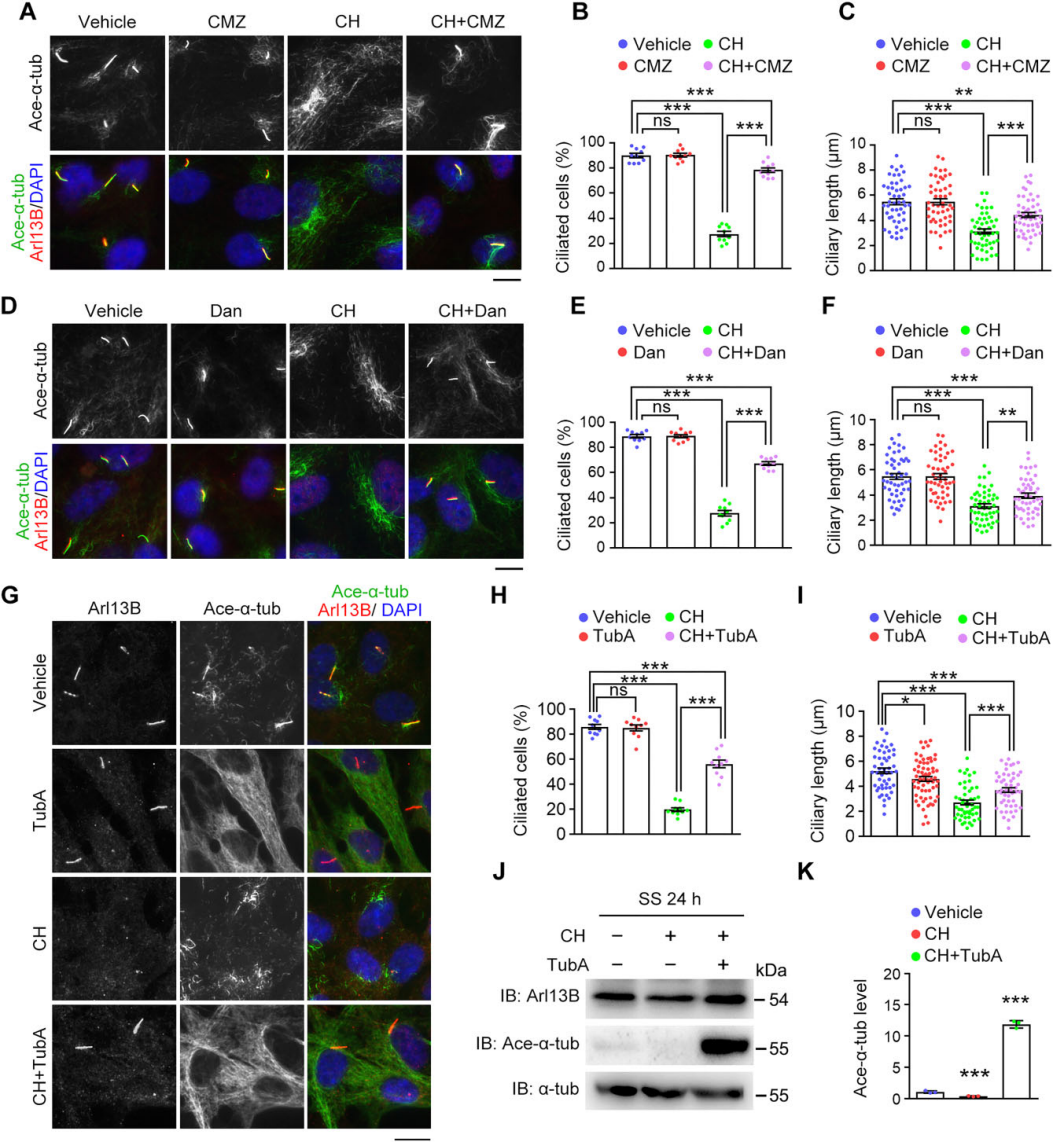
Aldehydes induce cilium disassembly via the calmodulin–Aurora A–HDAC6 axis. (**A**–**I**) RPE-1 cells were incubated with either vehicle (DMSO) or CMZ (2 μM; **A**–**C**), Dan (0.5 μM; **D**–**F**), or TubA (5 μM; **G**–**I**), in the presence or absence of CH (1 mM) treatment, in serum-free medium. (**A, D**, and **G**) Immunofluorescence images of ciliated cells stained for Arl13B, acetylated α-tubulin, and DAPI. Scale bar, 10 μm. (**B, E**, and **H**) The percentage of ciliated cells (*n* = 10 fields from three independent experiments). (**C, F**, and **I**) Quantification of ciliary length (*n* = 50 cilia from three independent experiments). (**J** and **K**) RPE-1 cells were incubated with vehicle (PBS), CH (1 mM), or CH (1 mM) plus TubA (5 μM) in serum-free medium. (**J**) Immunoblotting for Arl13B, α-tubulin, and acetylated α-tubulin. (**K**) Quantification of the level of acetylated α-tubulin (*n* = 3 independent experiments). Data are presented as mean ± SEM. An unpaired two-tailed *t*-test was performed. *P < 0.05; **P < 0.01; ***P < 0.001; ns, not significant.

Calcium influx is known to induce the binding of calmodulin to Aurora A, which in turn activates Aurora A to phosphorylate and stimulate HDAC6, resulting in axonemal microtubule deacetylation and destabilization (Sanchez and Dynlacht, 2016). We thus sought to test whether the calmodulin–Aurora A–HDAC6 pathway plays a role in aldehyde-induced cilium disassembly. Ciliated RPE-1 cells pretreated with calmidazolium chloride (CMZ), a calmodulin inhibitor (Plotnikova et al., 2012), or danusertib (Dan), a pan-inhibitor of the Aurora kinase family (consisting of Aurora A, Aurora B, and Aurora C) (Schoffski et al., 2015), were subjected to CH treatment. Immunostaining revealed that inhibition of either calmodulin or Aurora A rescued CH-induced ciliary defects (Figure 5A–F). We also used Aurora A-specific siRNAs to confirm these findings. As expected, siRNA-mediated depletion of Aurora A significantly inhibited CH-mediated cilium disassembly (Supplementary Figure S6A–D).

We then used tubastatin A (TubA), an HDAC6-specific inhibitor (Ran et al., 2022; Yang et al., 2022), to examine the role of HDAC6 in aldehyde-induced cilium disassembly. Ciliated RPE-1 cells pretreated with TubA were subjected to CH treatment. Similar to CMZ and Dan, TubA significantly prevented CH-induced cilium disassembly (Figure 5G–I). The inhibition of HDAC6 by TubA also dramatically elevated the level of acetylated α-tubulin in CH-treated cells (Figure 5J and K). In addition, similar to CH treatment (Figure 5J and K; Supplementary Figure S6E and F), glucose treatment promoted the phosphorylation of HDAC6 and reduced the level of cellular tubulin acetylation (Supplementary Figure S6G and H). Taken together, these results suggest that aldehydes cause microtubule deacetylation and destabilization via the calmodulin–Aurora A–HDAC6 pathway to induce cilium disassembly.

### Depletion of HDAC6 prevents acrolein-induced toxicity to cilia in mice

**Figure 6:**
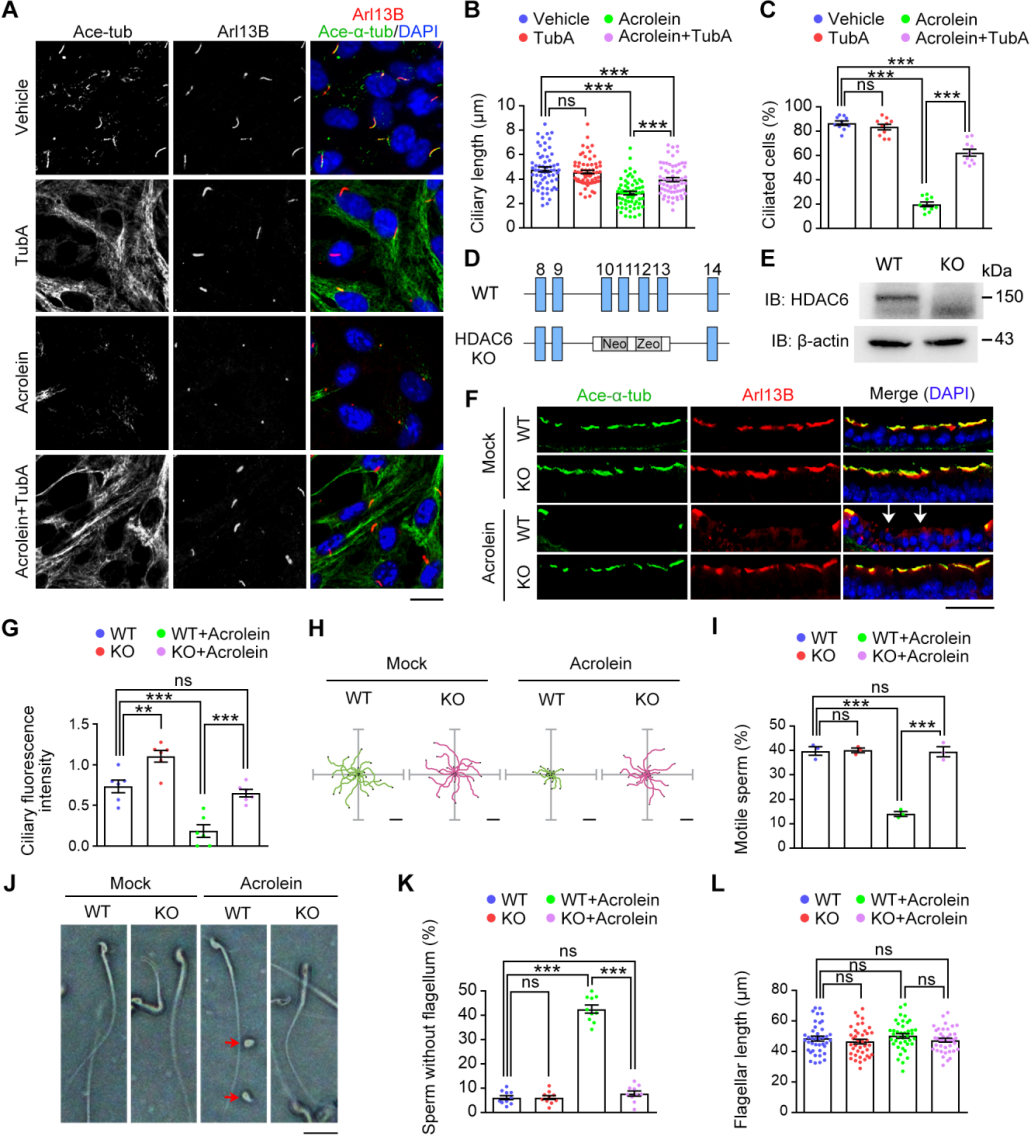
Loss of HDAC6 prevents cilium disassembly induced by acrolein exposure. (**A**–**C**) NIH-3T3 cells were incubated with vehicle (PBS), TubA (5 μM), acrolein (5 μM), or acrolein (5 μM) plus TubA (5 μM) in medium supplemented with 0.5% FBS. (**A**) Immunofluorescence images of ciliated cells stained for Arl13B, acetylated α-tubulin, and DAPI. Scale bar, 10 μm. (**B** and **C**) Quantification of ciliary length (*n* = 50 cilia from three independent experiments) and the percentage of ciliated cells (*n* = 10 fields from three independent experiments). (**D**) Strategy for the generation of *Hdac6* knockout mice. Insertion of a fragment containing neomycin and zeocin resistance genes (Neo and Zeo) results in a code-shifting mutation after exon 9 of the *Hdac6* gene. (**E**) Immunoblotting for HDAC6 in mouse tracheal tissues. (**F**–**L**) Wild-type (WT) and *Hdac6* knockout (KO) mice were either treated with acrolein for 2 weeks or untreated. (**F**) Immunofluorescence images of tracheal tissue sections stained for Arl13B, acetylated α-tubulin, and DAPI. Arrows indicate the loss of cilia in tracheal epithelial cells. Scale bar, 50 μm. (**G**) Quantification of ciliary fluorescence intensity in tracheal tissues (*n* = 6 mice). (**H**) Images of sperm movement trajectories for 3 sec (*n* = 20 sperm). The colored lines represent the movement tracks of sperm. Scale bar, 50 μm. (**I**) The percentage of sperm with normal motility (*n* = 3 independent experiments). (**J**) Images of sperm. The red arrows indicate two aberrant sperm with no flagella. Scale bar, 10 μm. (**K**) The percentage of aberrant sperm with no flagella (*n* = 3 mice). (**L**) Quantification of flagellar length of the remaining intact sperm (*n* = 50 sperm from 3 mice). Data are presented as mean ± SEM. An unpaired two-tailed *t*-test was performed. **P < 0.01; ***P < 0.001; ns, not significant.

Among the environmental aldehydes, acrolein is ubiquitously present in cooked foods, cigarette smoke, and automobile exhaust, and contact with acrolein may cause skin burns, erythema, and edema. The mechanism by which this common aldehyde produces toxic symptoms is still unknown. We found that treatment of cells with acrolein also led to a remarkable reduction in the percentage of ciliated cells and ciliary length (Figure 6A–C). In addition, inhibition of HDAC6 by TubA significantly blocked the deciliation induced by acrolein (Figure 6A–C).

To investigate the potential of targeting HDAC6 for the management of acrolein toxicity, we employed *Hdac6* knockout mice and confirmed the depletion of HDAC6 in these mice by immunoblotting (Figure 6D and E). Both wild-type and *Hdac6* knockout mice were treated with acrolein for 2 weeks and then compared with untreated mice. We found that the motile cilia of tracheal epithelial cells in wild-type mice were severely abrogated by acrolein treatment, while that in *Hdac6* knockout mice were well preserved after acrolein treatment (Figure 6F and G).

Acrolein has shown reproductive toxicity in the yeast spermatogenesis model (Golla et al., 2015). We found that sperm motility was significantly reduced in wild-type mice under acrolein stress but appeared normal in *Hdac6* knockout mice (Figure 6H and I). Notably, wild-type mice under acrolein treatment displayed a significantly increased proportion of aberrant sperm with no flagella, with the flagellar length of the remaining intact sperm unaffected, while acrolein treatment did not have any obvious effects on sperm morphology in *Hdac6* knockout mice (Figure 6J–L). Collectively, these results demonstrate a protective role for the depletion of HDAC6 against acrolein-induced toxicity to cilia.

## Discussion

CMDs are a group of diseases resulting from the dysregulation of carbohydrate metabolism and affect millions of people worldwide. CMDs cause severe complications in multiple organs, similar to those observed in ciliopathies. However, the underlying molecular mechanisms are obscure. In this study, we provide evidence that abnormal elevations of CMD-related aldoses, such as glucose, galactose, and mannose, rather than the corresponding sugar alcohols, lead to significant cilium disassembly. In addition, environmental aldehydes, such as formaldehyde, acetaldehyde, propionaldehyde, butyraldehyde, valeraldehyde, and benzaldehyde, are able to trigger cilium disassembly by the steric hindrance effect of their formyl groups. While the calmodulin–Aurora A–HDAC6 axis has been demonstrated to be the primary molecule cascade regulating cilium disassembly, our findings identify a previously unrecognized role for formyl group-mediated deciliation in the complications of CMDs.

Our live-cell imaging experiments show that aldehyde-induced calcium influx is critical for cilium disassembly. Calcium channels are known to mediate extracellular calcium influx across the plasma membrane, in particular voltage-gated calcium channels that mediate the rapid transport of extracellular calcium into the cytoplasm (Pitt et al., 2021). Voltage-gated calcium channels can be activated by various stimuli, such as membrane depolarization, signaling molecules, and physical forces acting on the plasma membrane (Cooper and Dimri, 2022). Aldehydes are organic compounds containing active terminal carbonyl groups, which are able to mediate covalent binding to amino and sulfhydryl groups of biomolecules, forming covalent linkage products, also known as adducts (LoPachin and Gavin, 2014). Since the plasma membrane consists of lipids and proteins, it is probably that the formyl groups of aldehydes may covalently bind to the amino or sulfhydryl groups of lipids and proteins, thereby compromising plasma membrane integrity and resulting in calcium influx across the plasma membrane.

The calcium–calmodulin complex has been reported previously to activate centrosomal Aurora A to trigger cilium disassembly (Plotnikova et al., 2012; Patel and Tsiokas, 2021). Consistent with this finding, our data show that aldehyde-induced cilium disassembly relies on the calmodulin–Aurora A signaling. In addition, our data demonstrate that the calmodulin–Aurora A signaling further activates HDAC6 to induce the deacetylation and destabilization of axonemal microtubules. However, it is noteworthy that cilium disassembly induced by aldehydes was only partially restored by inhibition of Aurora A or HDAC6, suggesting that additional mechanisms may contribute to aldehyde-induced cilium disassembly. For example, elevated calcium may activate other calcium-dependent protein kinases, such as protein kinase C, which subsequently lead to the phosphorylation of a variety of proteins to drive cilium disassembly (Zhu et al., 2011).

CH is an aldehyde widely used to remove both primary and motile cilia (Kennedy and Brittingham, 1968; Chakrabarti et al., 1998; Praetorius and Spring, 2003). The mechanisms by which aldehyde induces the loss of primary cilia remain elusive, although it has been reported to remove motile cilia by breaking down the axoneme from the basal body (Chakrabarti et al., 1998). Our data shown in this study suggest a cilium resorption model for the aldehyde-induced removal of primary cilia, which is different from the severing mechanism observed in motile cilia. In particular, by measuring the length of primary cilia at multiple time points after aldehyde treatment, we observed a gradual decrease in ciliary length over time, demonstrating that the cilia are undergoing resorption instead of severing. Considering the motile feature of motile cilia, it is plausible that the destabilized axonemal microtubules induced by aldehydes may be ‘forced’ to break down at the ciliary base for cilia continuously beating or rotating, driven by axonemal dynein motors. Further studies are warranted to examine the effect of aldehydes on motile cilia using a motility-deficient model.

Exposure to environmental aldehydes has many adverse effects on human health. Our data show that acrolein causes cilium disassembly not only in cultured cells but also in ciliated tissues, explaining the respiratory toxicity of inhaled acrolein. In addition, our data show that acetaldehyde, an intermediate metabolite of ethanol, induces cilium disassembly, providing novel insights into the harmful effects of long-term excessive alcohol consumption on the eyes, blood vessels, brain, liver, and fertility. Some aldehydes, such as benzaldehyde and vanillin, are used as food spices, but excessive doses may cause severe damage to the human body (Demir et al., 2010; Chen et al., 2012). Our data show that benzaldehyde induces cilium disassembly. It will be of great importance to investigate whether other flavoring aldehydes have similar effects to understand the toxicity of these agents. Using *Hdac6* knockout mice, we demonstrate that depletion of HDAC6 protects mice from aldehyde-induced cilium disassembly, suggesting that HDAC6 is a potential therapeutic target against aldehyde exposure.

## Materials and methods

### *Hdac6* knockout mice

*Hdac6* heterozygous mice (129/C57BL6 mixed genetic background) were kindly provided by Tso-Pang Yao (Duke University) and crossed to obtain homozygous knockout mice and wild-type mice. *Hdac6* knockout mice were fertile, and the male-to-female ratio in the offspring was 1:1. The mice used in our experiments were all males. Mice were housed in a temperature-controlled pathogen-free facility with a 12-h light/12-h dark cycle and provided with food and distilled water. All mouse experiments were carried out in accordance with the relevant regulatory standards and approved by the Animal Care and Use Committee of Nankai University.

### Cell culture

RPE-1 cells were obtained from the American Type Culture Collection and cultured in Dulbecco's modified Eagle medium/nutrient mixture F-12 (DMEM/F12) containing 10% fetal bovine serum (FBS; Biological Industries). NIH-3T3 cells were obtained from the American Type Culture Collection and cultured in DMEM supplemented with 10% FBS. All cells were cultured at 37°C with 5% CO_2_. To induce ciliogenesis, RPE-1 cells were cultured in serum-free medium for 24 h, and NIH-3T3 cells were cultured in medium with 0.5% FBS for 24 h. All the compounds used for cell culture are listed in Supplementary Table S1.

### Transfection of plasmids and siRNAs

Mammalian expression constructs for HA-tagged human HDAC6 were transfected into RPE-1 cells with Lipofectamine 3000 (Thermo Fisher Scientific) following the manufacturer's instructions. Two different siRNAs against Aurora A (#1, CCCUCAAUCUAGAACGCUA; #2, CGGUAGGCCUGAUUGGGUU) and the control siRNA (siN0000001-1) were purchased from RiboBio and transfected with Lipofectamine RNAiMAX (Thermo Fisher Scientific; Ran et al., 2021).

### Immunoprecipitation and immunoblotting

RPE-1 cells were lysed in lysis buffer (50 mM Tris–HCl, 150 mM NaCl, 1 mM EDTA, 3% glycerol, and 1% NP-40, pH 7.5) with a protease inhibitor cocktail (Thermo Fisher Scientific). To perform immunoprecipitation, cell lysates were incubated with anti-HA beads (Sigma-Aldrich, A2095) at 4°C for 4 h. The beads were washed 5 times with lysis buffer. Samples were denatured and separated by sodium dodecyl sulfate–polyacrylamide gel electrophoresis. The proteins were then transferred onto nitrocellulose membranes and blocked with 5% skim milk at room temperature for 2 h. The membranes were incubated with the primary antibody diluted in 5% skim milk at 4°C overnight, washed, and incubated with horseradish peroxidase-conjugated secondary antibody for 1 h at room temperature. Protein bands were visualized by using the luminol reagent (Millipore).

### Immunofluorescence microscopy

Immunostaining was performed as previously described (Ma et al., 2022). Briefly, cells grown on glass coverslips were fixed with ice-cold methanol for 3 min and then washed with phosphate-buffered saline (PBS). The cells were blocked with 4% bovine serum albumin (BSA) in PBS for 1 h at room temperature, incubated with the primary antibody at 4°C overnight, and then incubated with the secondary antibody for 1 h at room temperature. Coverslips were mounted onto slides with mounting medium containing DAPI (Solarbio). Mouse tracheal tissues were fixed overnight at 4°C in 4% paraformaldehyde, embedded in Tissue-Tek O.C.T. Compound (OCT; Sakura), and frozen in liquid nitrogen. The samples were sectioned at a thickness of 7 μm and then stored at −80°C. For immunostaining, tissue sections were washed with PBS to remove OCT and permeabilized using 0.5% Triton X-100 for 30 min. After incubation with 4% BSA for 1 h, tissue sections were stained following the procedure for staining cells. All the primary antibodies used are listed in Supplementary Table S2.

### Live-cell imaging

Ciliated RPE-1 cells were incubated in a solution of 2 μM Fluo-4 AM at 37°C for 30 min for fluorescent probe loading. Then, the cells were washed with PBS and immersed in serum-free medium for 30 min to ensure adequate conversion of Fluo-4 AM to Fluo-4 within the cells. Live-cell imaging was carried out on a Leica TCS SP5 confocal microscope with a 63× 1.4 NA oil objective at 37°C. Images were processed using ImageJ software (National Institutes of Health).

### Sperm analysis

Sperm were collected from the cauda epididymides isolated from sexually mature mice. Briefly, cauda epididymides were dissected to remove adipose tissue, immersed in PBS, and cut into 4–6 segments using straight iris scissors. Sperm were released from the segments into PBS by incubation at 37°C for 15 min. To track sperm motility, a 5-sec video of sperm swimming was recorded using an Olympus microscope equipped with a Canon camera. The video was subsequently converted into 10 images per second and analyzed with ImageJ software with the manual tracking and chemotaxis-migration tool plugins as described (Zhang et al., 2016). The length of the sperm flagellum was also measured with ImageJ.

### Statistical analysis

Statistical analysis was performed using Prism software (GraphPad). Statistical results are shown as mean ± SEM. Significant differences between two groups were analyzed using an unpaired two-tailed *t*-test.

## Supplementary Material

mjad079_Supplemental_Files

## Notes

**Author contributions:** J.Z., H.Z., and M.L. designed and supervised this study. T.L., W.B., and K.L. conducted the experiments and analyzed the data. M.L., F.Y., and S.Y. provided practical and technical assistance. K.L., J.Y., H.N., M.Y., H.Y., and R.H. conducted the animal experiments. J.Z., H.Z., and D.L. provided constructive discussions. T.L., J.Z., and H.Z. wrote the manuscript with input from all other authors.
