# Supplementary material for: Pathologically relevant aldoses and environmental aldehydes cause cilium disassembly via formyl group-mediated mechanisms
Source: J Mol Cell Biol. 2023 Dec 6;16(1):mjad079. doi: 10.1093/jmcb/mjad079 (PMC11245732; doi:10.1093/jmcb/mjad079)
Supplement: mjad079_Supplemental_Files [file mjad079_supplemental_files.zip › Supplementary figures.pdf]

Supplementary Data

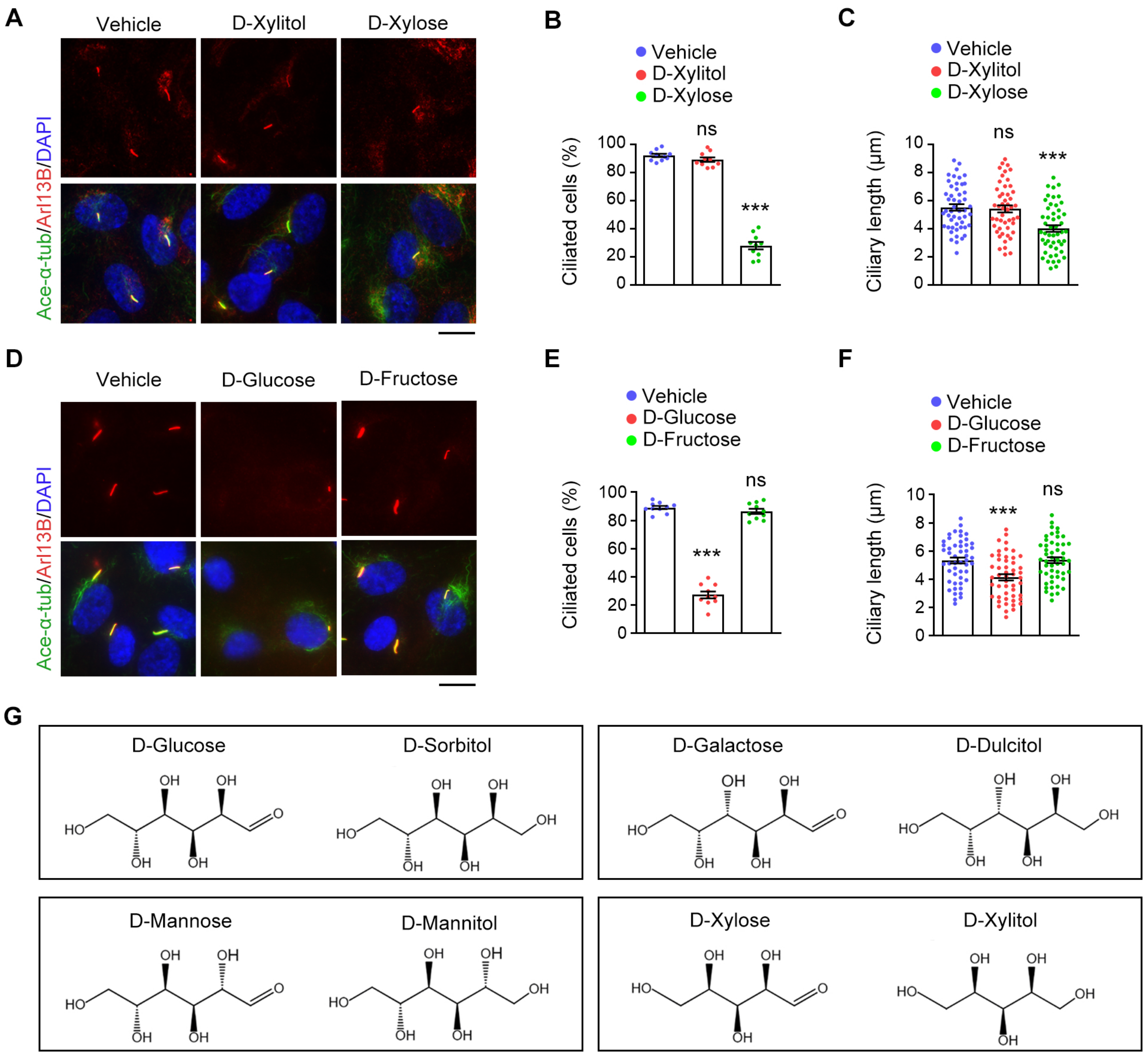

Figure S1 Distinct effects on cilium disassembly and structural differences between aldoses and sugar alcohols. (A-F) Immunofluorescence images (A and D) and quantification of the percentage of ciliated cells (B and E; n = 10 fields from 3 independent experiments) and ciliary length (C and F; n = 50 cilia from 3 independent experiments) for RPE-1 cells incubated with vehicle (PBS) or the indicated chemicals (25 mM) in serum-free medium. RPE-1 cells were stained with antibodies against Arl13B and acetylated α-tubulin, and DAPI. scale bar, 10 μm. **(G)** Chemical structures of the indicated aldoses and corresponding sugar alcohols. Data are from three independent biological repeats and presented as mean ± SEM. An unpaired two-tailed t-test was performed. P < 0.001 (***); ns, not significant.

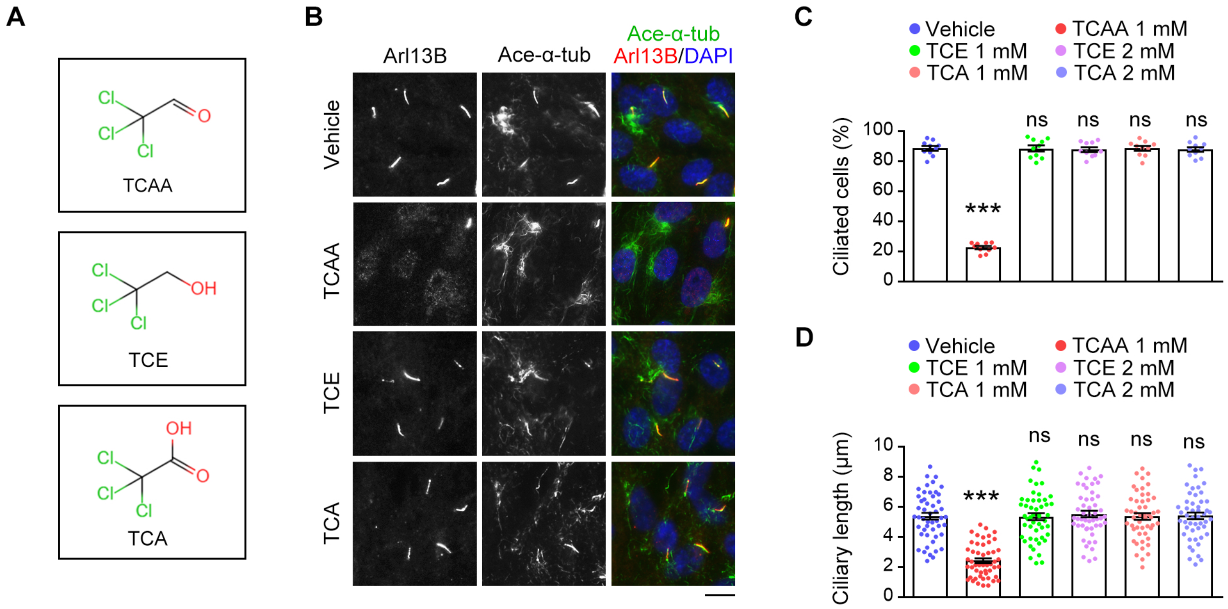

Figure S2 Different effects of trichloroacetaldehyde (TCAA), trichloroethanol (TCE), and trichloroacetic acid (TCA) on cilium disassembly. (A) Chemical structures of TCAA, TCE, and TCA. **(B-D)** Immunofluorescence images (B) and quantification of the percentage of ciliated cells (C; n = 10 fields from 3 independent experiments) and ciliary length (D; n = 50 cilia from 3 independent experiments) for RPE-1 cells incubated with vehicle (PBS), 1 mM TCAA, 1 mM TCE, 2 mM TCE, 1 mM TCA, or 2 mM TCA in serum-free medium. RPE-1 cells were stained with antibodies against Arl13B and acetylated α-tubulin, and DAPI. In panel B, representative images showing the effects of the compounds at 1 mM are shown. Scale bar, 10 μm. Data are from three independent biological repeats and presented as mean ± SEM. An unpaired two-tailed t-test was performed. ns, not significant.

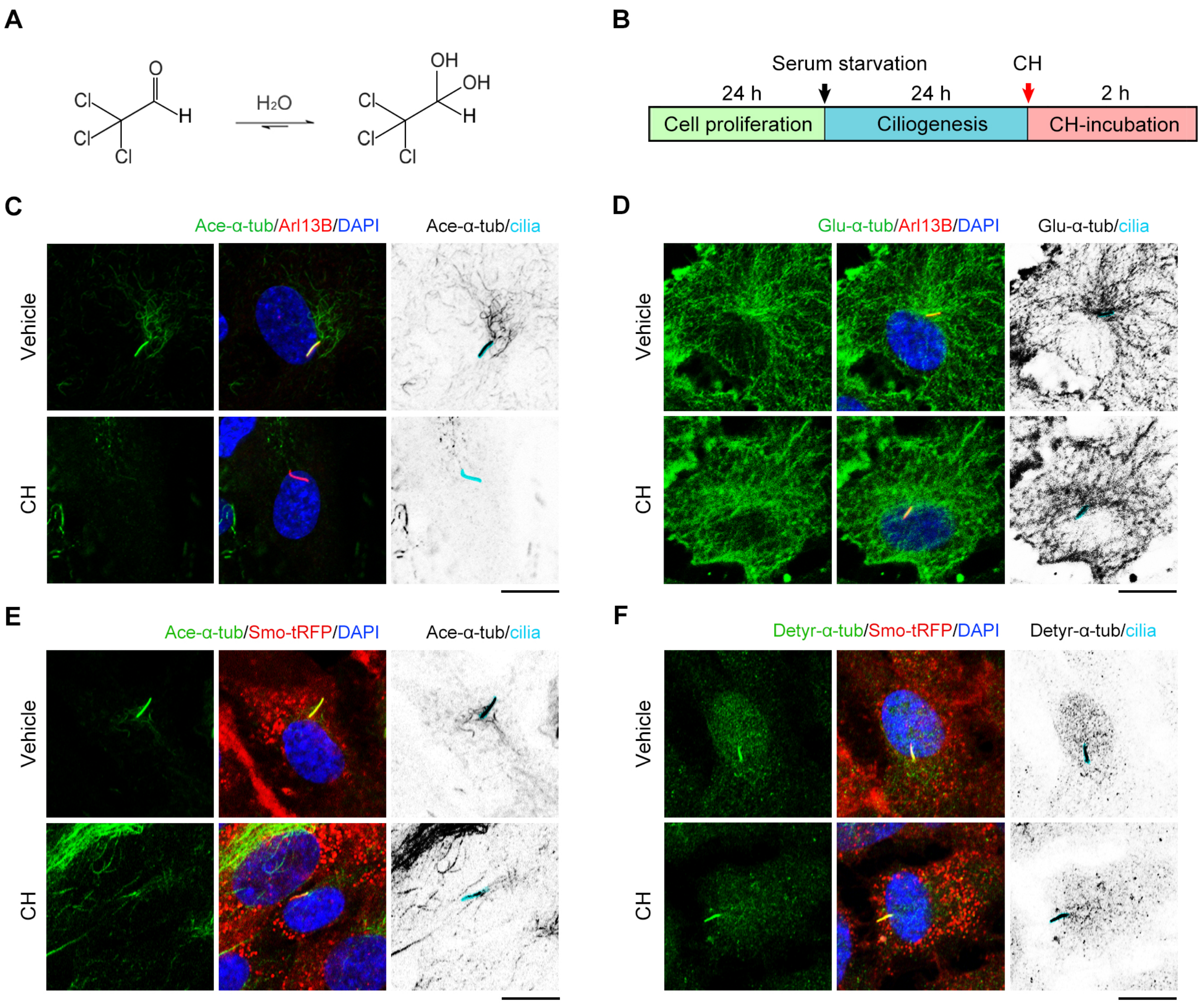

Figure S3 Different effects of CH treatment on post-translational modifications of axonemal microtubules. (A) Trichloroacetaldehyde reacts with water to form CH. **(B)** Strategy used to study the effects of CH treatment on post-translational modifications of axonemal microtubules. Ciliated RPE-1 cells were serum starved for 12 h to induce cilium formation, followed by the treatment of 25 mM CH for 2 h. **(C, D)** Immunofluorescence images of RPE-1 cells incubated with vehicle (PBS) or CH (25 mM) in serum-free medium. RPE-1 cells were stained with antibodies against Arl13B and acetylated α-tubulin (C) or polyglutamylated α-tubulin (D), and DAPI. **(E, F)** Immunofluorescence images of Smo-tRFP expressing RPE-1 cells incubated with vehicle (PBS) or CH (25 mM) in serum-free medium. Cells were stained with antibodies against acetylated α-tubulin (E) or detyrosinated α-tubulin (F), and DAPI. Scale bar, 10 μm.

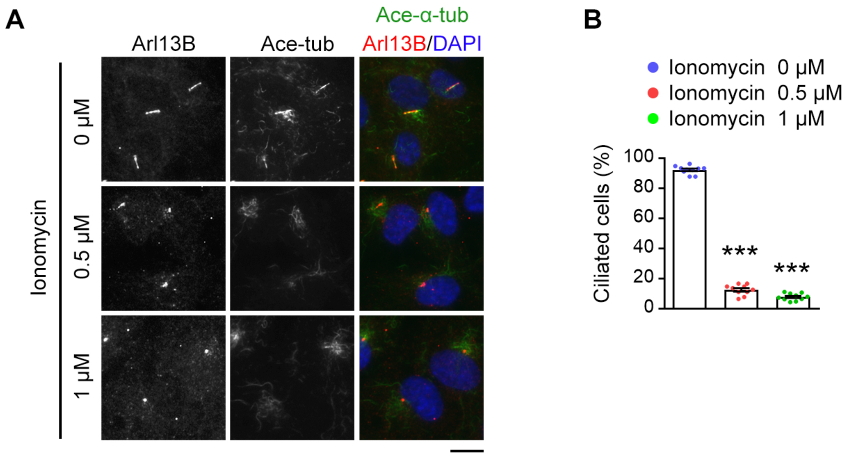

Figure S4 Ionomycin induces cilium disassembly. (A, B) Immunofluorescence images (A) and quantification of the percentage of ciliated cells (B; n = 10 fields from 3 independent experiments) for RPE-1 cells incubated with vehicle (DMSO) or ionomycin in serum-free medium. RPE-1 cells were stained with antibodies against Arl13B and acetylated α-tubulin, and DAPI. Scale bar, 10 μm. Data are from three independent biological repeats and presented as mean ± SEM. Unpaired two-tailed t-test was performed. P < 0.001 (***).

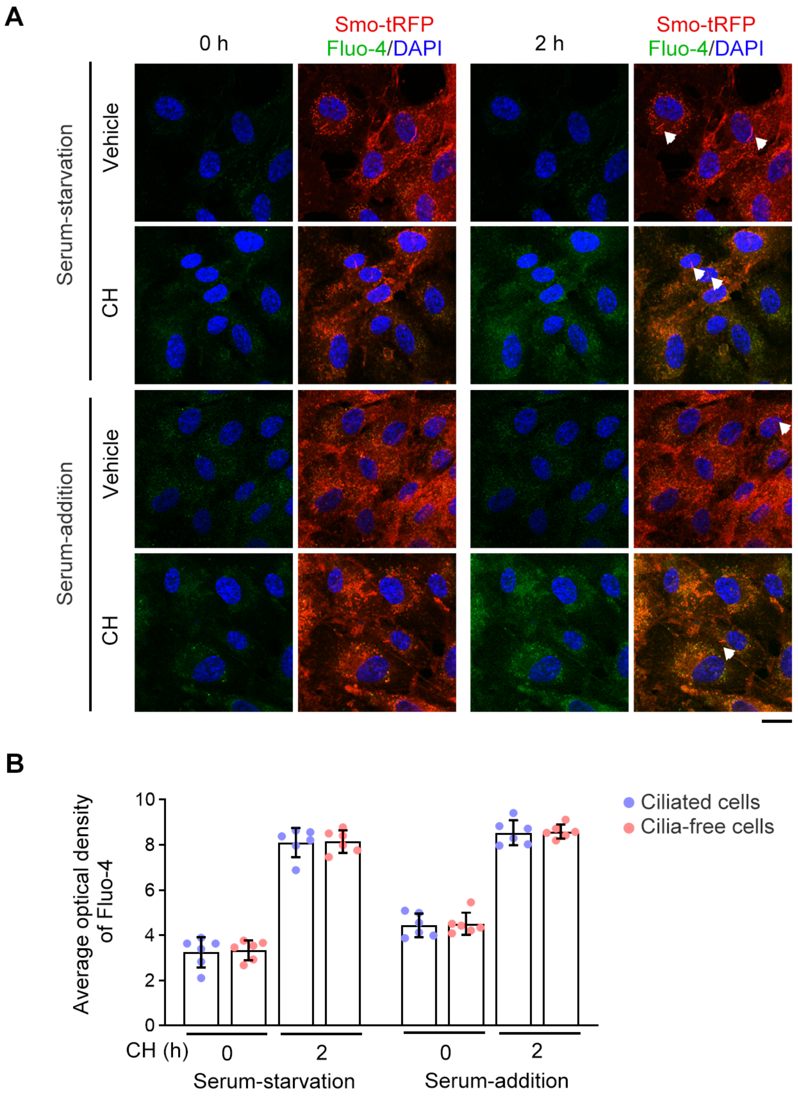

Figure S5 Aldehyde-induced activation of calcium signals is independent of cilia. (A, B) Immunofluorescence images (A) and quantification of the percentage of ciliated cells (B; n = 6 cells from an experiment). Smo-tRFP expressing RPE1- cells were serum starved for 12 h to induce cilium formation, followed by the treatment of vehicle (PBS) or CH (25 mM) in serum-free or serum-containing medium. Cells were stained with DAPI. Scale bar, 10 μm. Data are presented as mean ± SEM.

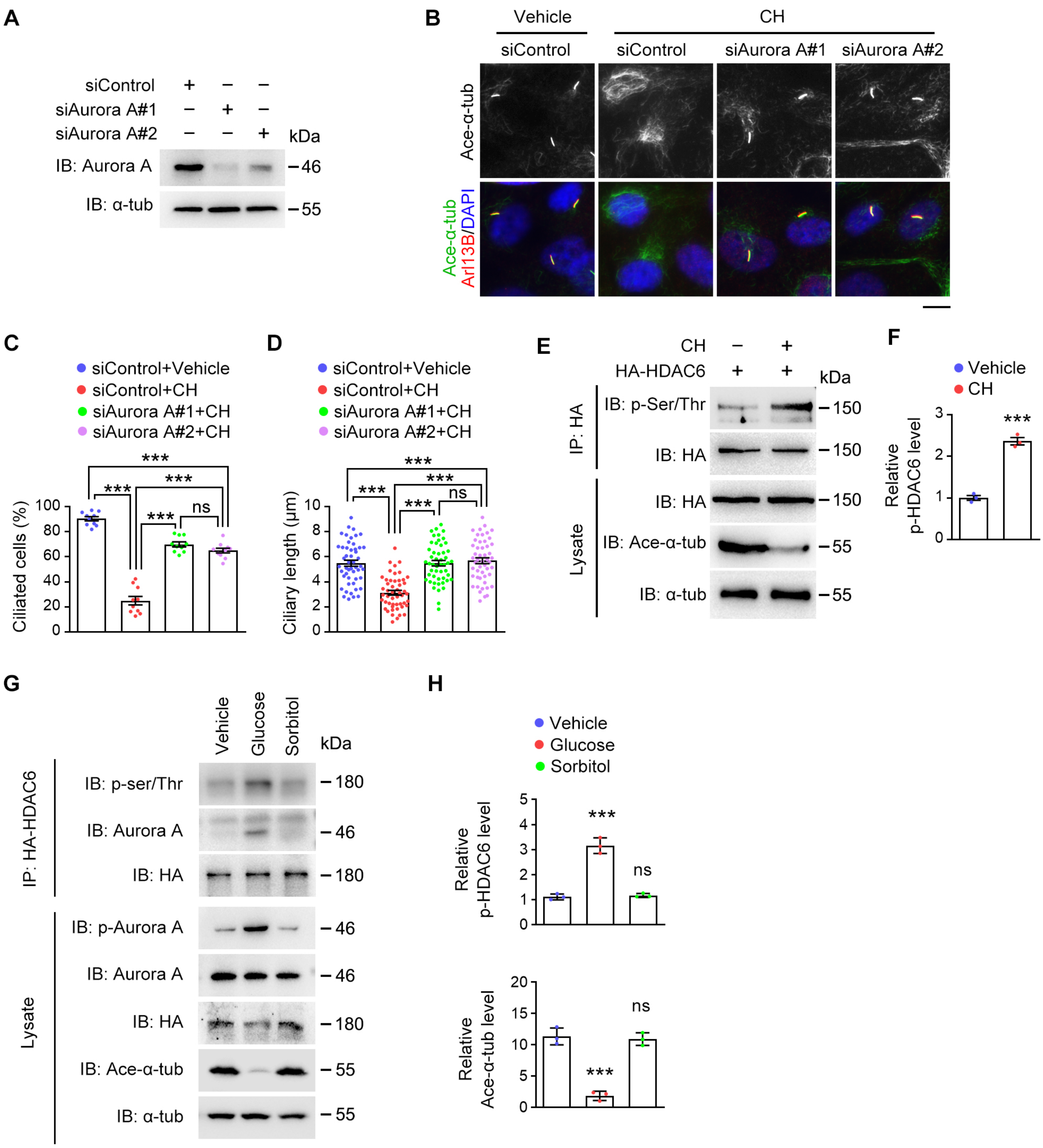

Figure S6 Involvement of Aurora A and HDAC6 in CH-mediated cilium disassembly. (A) Immunoblot analysis of Aurora A and α-tubulin in RPE-1 cells transfected with control or Aurora A siRNAs. **(B-D)** Immunofluorescence images (B) and quantification of the percentage of ciliated cells (C; n = 10 fields from 3 independent experiments) and ciliary length (D; n = 50 cilia from 3 independent experiments) for RPE-1 cells transfected with the indicated siRNAs and incubated with vehicle (PBS) or CH (1 mM) in serum-free medium. RPE-1 cells were stained with antibodies against Arl13B and acetylated α-tubulin, and DAPI. Scale bar, 10 μm. **(E, F)** Immunoblot analysis (E) and quantification (F; n = 3 independent experiments) of HDAC6 phosphorylation in RPE-1 cells transfected with HA-HDAC6 with or without CH treatment. The intensity of the phospho-Ser/Thr band was normalized to that of the HA-HDAC6 band. **(G, H)** Immunoblot analysis (G) and quantification (H; n = 3 independent experiments) of HDAC6 phosphorylation in RPE-1 cells transfected with HA-HDAC6 with 25 mM glucose or sorbitol treatment. The intensity of the phospho-Ser/Thr band was normalized to that of the HA-HDAC6 band. Data are from three independent biological repeats and presented as mean ± SEM. Unpaired two-tailed t-test was performed. P < 0.001 (***); ns, not significant.
